# Accurate Electron Affinity of Iron and Fine Structures of Negative Iron ions

**DOI:** 10.1038/srep24996

**Published:** 2016-05-03

**Authors:** Xiaolin Chen, Zhihong Luo, Jiaming Li, Chuangang Ning

**Affiliations:** 1 Department of Physics, State Key Laboratory of Low-Dimensional Quantum Physics, Tsinghua University, Beijing 10084, China; 2 Collaborative Innovation Center of Quantum Matter, Beijing, China

## Abstract

Ionization potential (IP) is defined as the amount of energy required to remove the most loosely bound electron of an atom, while electron affinity (EA) is defined as the amount of energy released when an electron is attached to a neutral atom. Both IP and EA are critical for understanding chemical properties of an element. In contrast to accurate IPs and structures of neutral atoms, EAs and structures of negative ions are relatively unexplored, especially for the transition metal anions. Here, we report the accurate EA value of Fe and fine structures of Fe^−^ using the slow electron velocity imaging method. These measurements yield a very accurate EA value of Fe, 1235.93(28) cm^−1^ or 153.236(34) meV. The fine structures of Fe^−^ were also successfully resolved. The present work provides a reliable benchmark for theoretical calculations, and also paves the way for improving the EA measurements of other transition metal atoms to the sub cm^−1^ accuracy.

Iron is the second most abundant metal element on earth. It is an essential part of human being^1^, which is central to the structure and functioning of blood in transporting oxygen around the body. For over three thousand years, iron formed the material basis of human civilization. The Iron Age is after the Bronze Age in the three pre-historical ages due to the ease of corrosion and the relatively high melting point of iron. Nowadays, steel, an iron based material, is one of the most common materials in world. Iron and iron compounds are magnetic^2^. They can also be used as catalysis^3^. Recently, some iron based materials were reported as a new class of superconductors^4^,^5^. These fantastic properties of iron are directly related to its unique electronic structures. However, it is still a challenge to fully understand them. Even for the single negative atomic ion, Fe^−^, it is a nontrivial task for both experimental and theoretical investigation^6^,^7^,^8^,^9^,^10^,^11^,^12^,^13^,^14^,^15^,^16^. The properties of negative ions differ significantly from both positive ions and neutral systems^17^,^18^,^19^,^20^. Like the ionization potential, the electron affinity (EA) is a fundamental parameter for understanding chemical properties of elements^21^,^22^. The detailed knowledge of fine structures of anions is also required by laser cooling of negative ions^23^,^24^,^25^,^26^,^27^,^28^.

Electron affinities of atoms and molecules are mainly measured by photoelectron spectroscopy of negative ions, A^−^ + *hν* → A + e, and EA = *hν* − *E*_*k*_. *hν* is the photon energy, and *E*_*k*_ is the kinetic energy of photoelectrons. The EA value of Fe, 164(35) meV, was first reported by Engelking and Lineberger in 1979^6^. Then, it was improved to 151(3) meV by Leopold and Lineberger in 1986^7^. After their pioneering work, no significant improvement has been reported during the past 30 years. On the other side, the accuracy of experimental EA value for some transition metal elements and main group elements have been steadily improved to 0.01–0.05 meV^29^,^30^,^31^. The EA uncertainty by s-wave photodetachment even goes down to 1 μeV level by using the laser photodetachment microscopy^32^,^33^,^34^,^35^,^36^. Most of the accurate EA values for transitional metals, such as EA(Cu) = 1235.78(4) meV^37^, were obtained by the laser photodetachment threshold (LPT) method^38^. LPT measures the photodetachment cross section versus the photon energy around the photodetachment threshold using the narrow linewidth tunable laser. The outgoing photoelectron is a p-wave for the threshold photodetachment from atomic transition metal anions. Therefore, the photodetachment cross section near the threshold is very small, according to the Wigner threshold law^39^. Usually, the LPT method requires a strong anion beam and a high-intensity laser beam. However, it is difficult to produce an intense Fe^−^ ion beam due to its low EA value. Furthermore, the EA measurement of Fe using LPT method requires a tunable light source in the far infrared band, which is a luxury experimental apparatus. Moreover, LPT method cannot well resolve the congested photodetachment channels due to the zero-slope onset of p-wave detachment at threshold^39^. As shown later, the ability to resolve the congested photodetachment channels is crucial to measure the fine structures of Fe^−^. Similar to the case of Fe, the uncertainties of EA values for many other transition metals also remain 10 meV^40^,^41^,^42^. The experiment method we demonstrated in this study can serve as a powerful approach to improve the EA measurement and fine structure for other transition metal elements.

In this study, the accurate EA value of Fe and the fine structures of Fe^−^ were obtained using the slow electron velocity imaging (SEVI) method. SEVI has a super energy resolution for slow electrons^43^,^44^,^45^,^46^. Recently, an energy resolution 1.2 cm^−1^ for *E*_k_ = 5.2 cm^−1^ has been reported by Wang and coworkers^45^. The conversion factor between cm^−1^ and eV is 1 eV = 8065.544 005(50) cm^−1^, recommended by CODATA^47^. Since the neutral Fe atomic energy levels are well known, this gives a freedom to choose the final neutral state of photodetachment. This flexibility is crucial for current Fe study and other elements with a low EA value^48^. To avoid using a tunable laser in far infrared region, the Fe(^3^F_4_) ← Fe^−^(^4^F_9/2_) channel, threshold photodetachment wavelength λ ≈ 745 nm, is chosen for conducting the Fe EA measurement. With our newly constructed SEVI apparatus, this study successfully resolved all fine structures of Fe^−^ and significantly improved the EA accuracy of Fe.

## Results

### Photoelectron Spectroscopy

**Figure 1:**
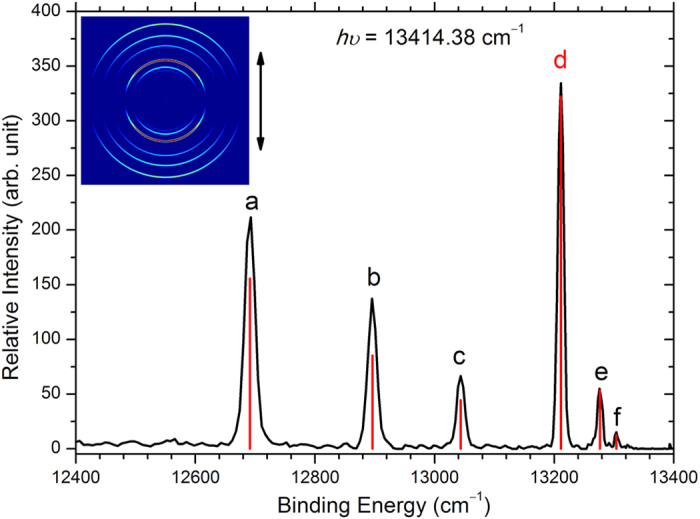
Photoelectron image and spectrum of Fe^−^ at photodetachment energies 13414.38 cm^−1^. The double arrow indicates the laser polarization. Peak d is a result of photodetachment from Fe^−^(^4^F_9/2_) to Fe(^3^F_4_), which is used to determine the electron affinity of Fe. The vertical red spikes are the theoretical simulations at the ion temperature 800 K.

**Figure 2:**
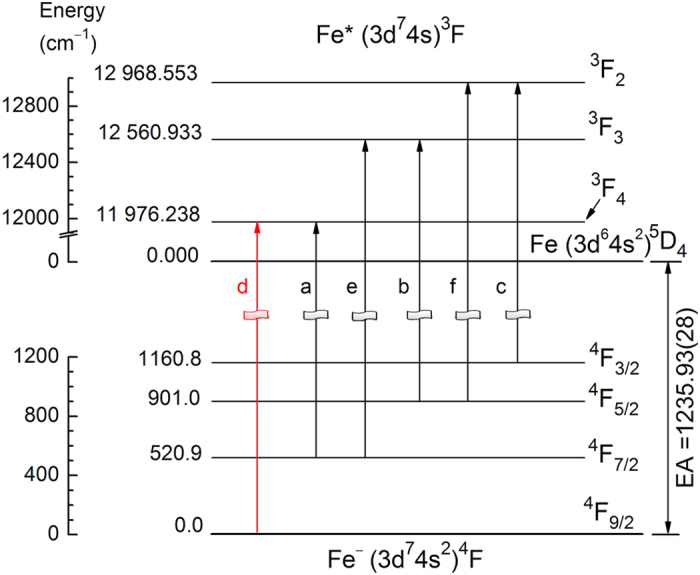
Energy levels of Fe and Fe^−^ related to the present measurement. The ground state of Fe is 3d^6^4s^2 5^D_4_. The ground state of Fe^−^ is 3d^7^4s^2 4^F_9/2_. The labels of each transition are the indexes of the observed peaks in Fig. 1. The transition d is used for the electron affinity measurement.

The descriptions of our spectrometer have been reported in previous work^49^. The Fe^−^ ion beam was produced by a laser ablation ion source assisted with sodium vapor. The photoelectron spectra were obtained for Fe^−^ at various detachment laser wavelengths. Figure 1 presents the spectrum at a photon energy *hν* = 13414.38 cm^−1^. There are six sharp peaks labelled with letters (a–f). The photoelectron imaging shown in the inset clearly shows expected parallel transitions due to the p-wave detachment. The related transitions of each peak are shown in Fig. 2. The vertical spikes in Fig. 1 are the theoretical simulation according to the assigned transitions. These intensity simulations were derived by assuming the ion temperature of 800 K^6^ and further rescaled according to the Wigner threshold law σ ∝ *E*_k_^3/2^ for p-wave detachment. Here σ is the cross section of photodetachment. The excellent agreement between experimental results and simulations confirmed the validity of current assignment. Based on the assignment, transition d [Fe(^3^F_4_) ← Fe^−^(^4^F_9/2_)] is the only photodetachment channel originated from the ground state Fe^−^(^4^F_9/2_), so it was selected as the target channel for the accurate EA measurement.

### Electron Affinity and Fine Structures

**Figure 3:**
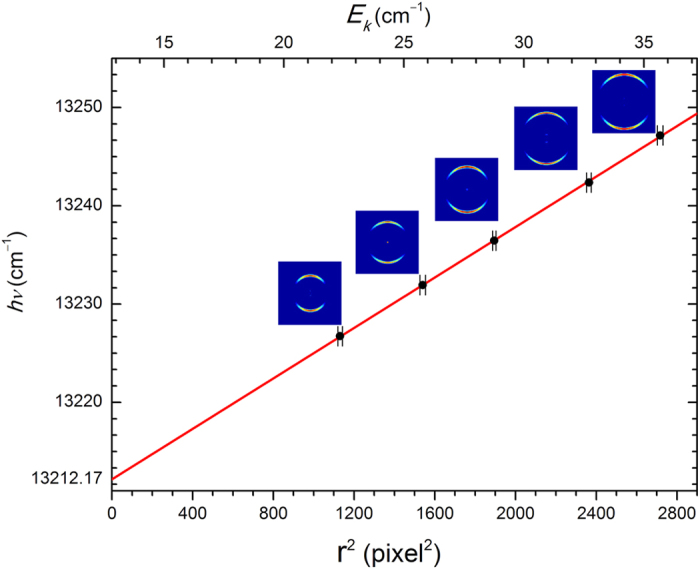
Energy calibration of the photoelectron imaging system. Points with error bars are experimental data. The solid line is the best linear fitting. The rings above each point are the photoelectrons imaging of Fe(^3^F_4_) ← Fe^−^(^4^F_9/2_). The ring radius *r* is in unit of pixel.

**Figure 4:**
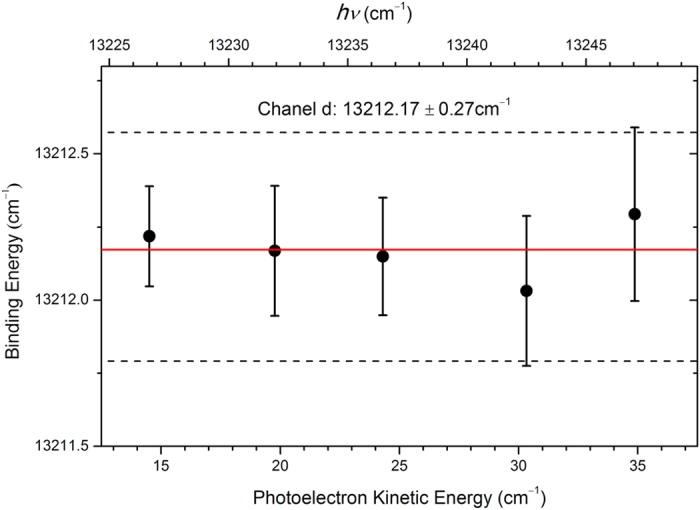
Binding energy of Fe(^3^F_4_) ← Fe^−^(^4^F_9/2_) transition measured as a function of the kinetic energy of photoelectrons. The dotted lines indicate the ±0.27 cm^−1^ uncertainty.

In order to obtain high accurate EA of Fe, the photoelectron imaging system for the transition d was carefully calibrated. After inverse-Abel transformation^50^, the hitting positions of photoelectrons on the phosphor screen form a ring for each individual transition. The radius *r* of the ring is proportional to the velocity of photoelectrons. The radius can be obtained by summing the intensity over all angles, and then finding the peak center via a Gaussian profile fitting. A series of photoelectron spectra were measured with the photon energy scanned from 13227 cm^−1^ to 13247 cm^−1^ with a step 5 cm^−1^. The measured radius square (*r*^2^) of transition d versus the photon energy *hν* was plotted in Fig. 3. The energy calibration parameters of the linear relation between *hν* and *r*^2^ were determined by linear fitting. The binding energy of transition d and its uncertainty can be also derived from this procedure. Figure 4 shows the measured binding energy versus the photoelectron kinetic energy. The mean binding energy is 13212.17 cm^−1^ with an uncertainty 0.27 cm^−1^. The neutral Fe (^3^F_4_) state is 11976.239 cm^−1^ above the iron neutral ground state (^5^D_4_). Therefore, EA(Fe) is determined as 1235.93 ± 0.28 cm^−1^. The uncertainty 0.28 cm^−1^ has included the 0.06 cm^−1^ laser linewidth.

The fine structure of Fe^−^(^4^F) were derived from the observed transitions. The splitting of Fe^−^(^4^F_7/2_) ← Fe^−^(^4^F_9/2_), Fe^−^(^4^F_5/2_) ← Fe^−^(^4^F_9/2_), Fe^−^(^4^F_3/2_) ← Fe^−^(^4^F_9/2_) was determined as 520.9(11), 901.0(14), 1160.8(15) cm^−1^ by the standard spectroscopic method, the covariance algebra, respectively^51^,^52^.

The measured binding energies of transitions and fine structures of Fe^−^ are summarized in Tables 1, 2, 3. The measured fine structures of Fe^−^ are in comparison with the calculated ones using the spin-orbit coupling multi-reference configuration interaction method. The calculated values are slightly higher than the experimental results. The measured EAs were also compared with the theoretical predictions in Tables 1, 2, 3. The small value of EA of iron is a particular challenge to theory. Some methods even predicted a negative binding energy.

The energy gaps between different neutral Fe states can also be extracted from the six transitions. It is worth comparing them with the standard atomic data^53^. The interval between peaks d and e is 585.4 cm^−1^, in an excellent agreement with the energy difference 584.695 cm^−1^ between ^3^F_3_ and ^3^F_4_ states of neutral Fe. Similarly, we have an energy interval 408.3 cm^−1^ between peaks b and f versus 407.620 cm^−1^ between Fe(^3^F_2_) and Fe(^3^F_3_). These accurate data can be considered as the fingerprints of anionic states for the unambiguous assignment.

## Discussion

In conclusion, the EA value of Fe was determined as 1235.93(28) cm^−1^ or 153.236(34) meV using the slow electron velocity imaging method. The accuracy of EA of Fe was improved by a factor more than 80 compared with previous reported 151(3) meV^7^. The fine structures of Fe^−^ were successfully resolved. The Fe^− 4^F_7/2_, ^4^F_5/2_, ^4^F_3/2_ are 520.9(11) cm^−1^, 901.0(14) cm^−1^, and 1160.8(15) cm^−1^ above the ground state ^4^F_9/2_, respectively.

During past 40 years, the measurement accuracy of the electron affinity (EA) of main group elements has been steadily improved to 0.01–0.05 meV. However, the uncertainties of EA values of many transition elements still remain 10 meV^40^,^41^,^42^. The experimental EA values for most of the f-block lanthanides and actinides are not available yet^54^,^55^,^56^,^57^. The super energy resolution of SEVI method combined with the sodium vapor assisting laser ablation ion source makes it possible to improve the EA measurement accuracy to sub cm^−1^ for nearly all transition metal atoms.

## Methods

The experiment was conducted using a slow electron velocity imaging apparatus equipped a laser ablation ion source. The Fe^−^ ion beam was produced by a laser ablation ion source. Sodium vapor was introduced to enhance Fe^−^ yield by an inline oven. The^56^ Fe^−^ ions were selected by a Wiley-McLaren type time-of-flight mass spectrometer. Then, the selected ions were perpendicularly crossed by the detachment laser beam in the interaction zone. The photodetachment laser is from a Spectra-physics dye laser system (400–920 nm, line width 0.06 cm^−1^ at 625 nm) pumped by a Quanta-Ray Pro 290 Nd:YAG laser (20 Hz, 1000 mJ/pulse at 1064 nm). The photon energy (*hν*) was further measured by a HighFinesse WS6-600 wavelength meter with an accuracy of 0.02 cm^−1^. The detached photoelectrons were projected onto a phosphor screen behind a set of micro-channel plates and recorded by a CCD camera. Each photoelectron imaging was an accumulated result of 200,000 laser shots. The photoelectron spectrum was then generated by an inverse Abel transformation of the raw photoelectron imaging. The obtained energy resolution is 3.1 cm^−1^ for *E*_k_ = 25 cm^−1^ at an imaging voltage −150 V. It should be noted that the energy resolution Δ*E*_k_ depends on the kinetic energy *E*_k_, roughly Δ*E*_k_ ∝ *E*_k_^1/2^.

The fine structures of Fe^−^ were calculated using the spin–orbit coupling multireference configuration interaction method with the TZP-DKH basis set. The TZP-DKH basis set was obtained from the basis set exchange website https://bse.pnl.gov. The calculations were carried out using the Molpro package.

## Additional Information

**How to cite this article**: Chen, X. *et al.* Accurate Electron Affinity of Iron and Fine Structures of Negative Iron ions. *Sci. Rep.* **6**, 24996; doi: 10.1038/srep24996 (2016).

## Figures and Tables

**Table 1:** Measured binding energies, fine structures of Fe^−^, and the electron affinity.

Peak	Levels (Fe ← Fe^−^)	Binding energy (cm^−1^)
a	^3^F_4_ ← 4F_7/2_	12691.1(11)
b	^3^F_3_ ← 4F_5/2_	12895.8(14)
c	^3^F_2_ ← 4F_3/2_	13043.7(14)
d	^3^F_4_ ← 4F_9/2_	13212.17(28)
e	^3^F_3_ ← 4F_7/2_	13276.5(21)
f	^3^F_2_ ← 4F_5/2_	13304.1(34)

**Table 2:** Fine structure of Fe^−^ (cm^−1^).

Levels	Calculated/extrapolated	Experimental
^4^F_7/2_ ← ^4^F_9/2_	543/540(50)^41^	520.9(11)
^4^F_5/2_ ← ^4^F_9/2_	965/930(60)^41^	901.0(14)
^4^F_3/2_ ← ^4^F_9/2_	1267/1200(60)^41^	1160.8(15)

**Table 3:** The electron affinity of Fe and references.

Value (meV)	Reference
580	Clementi^8^ (calculated)
−220	Cole *et al.*^9^ (calculated)
−30	Mitas^10^(calculated)
210	Bauschlicher *et al.*^11^ (calculated)
−110	Buendia *et al.*^12^ (calculated)
78	Balabanov *et al.*^14^ (calulated)
164 (35)	Engelking *et al.*^6^ (measured)
151 (3)	Leopold *et al.*^7^ (measured)
153.236(34)	this work (measured)
